# Development and scale-up of a commercial fed batch refolding process for an anti-CD22 two chain immunotoxin

**DOI:** 10.1002/btpr.1983

**Published:** 2014-09-02

**Authors:** Thomas Linke, Matthew T Aspelund, Christopher Thompson, Guoling Xi, Andrew Fulton, Michaela Wendeler, Timothy M Pabst, Xiangyang Wang, William K Wang, Kripa Ram, Alan K Hunter

**Affiliations:** Dept. of Purification Process Sciences, MedImmune, One MedImmune WayGaithersburg, MD, 20878

**Keywords:** moxetumomab pasudotox, scale-up, immunotoxin, inclusion body, refolding

## Abstract

We describe the development and scale-up of a novel two chain immunotoxin refolding process. This work provides a case study comparing a clinical manufacturing process and the commercial process developed to replace it. While the clinical process produced high quality material, it suffered from low yield and high yield variability. A systematic approach to process development and understanding led to a number of improvements that were implemented in the commercial process. These include a shorter inclusion body recovery process, limiting the formation of an undesired deamidated species and the implementation of fed batch dilution refolding for increased refold titers. The use of a combination of urea, arginine and DTT for capture column cleaning restored the binding capacity of the capture step column and resulted in consistent capture step yields compared to the clinical process. Scalability is shown with data from 250 L and 950 L scale refolding processes. Compared to the clinical process it replaces, the commercial process demonstrated a greater than fivefold improvement in volumetric productivity at the 950 L refolding scale. © 2014 American Institute of Chemical Engineers *Biotechnol. Prog*., 30:1380–1389, 2014

## Introduction

Recombinant immunotoxins represent an important class of anticancer drugs typically composed of truncated protein toxins fused to an antibody fragment.^1^–^4^ The antibody fragment replaces the cell-binding domain of the native toxin, allowing the toxin molecule to be directed against an oncology target of interest. Toxins evaluated in the clinic as part of immunotoxin therapy trials include those derived from ricin, diphtheria toxin, and *Pseudomonas aeruginosa* exotoxin A (PE).^3^

**Figure 1:**
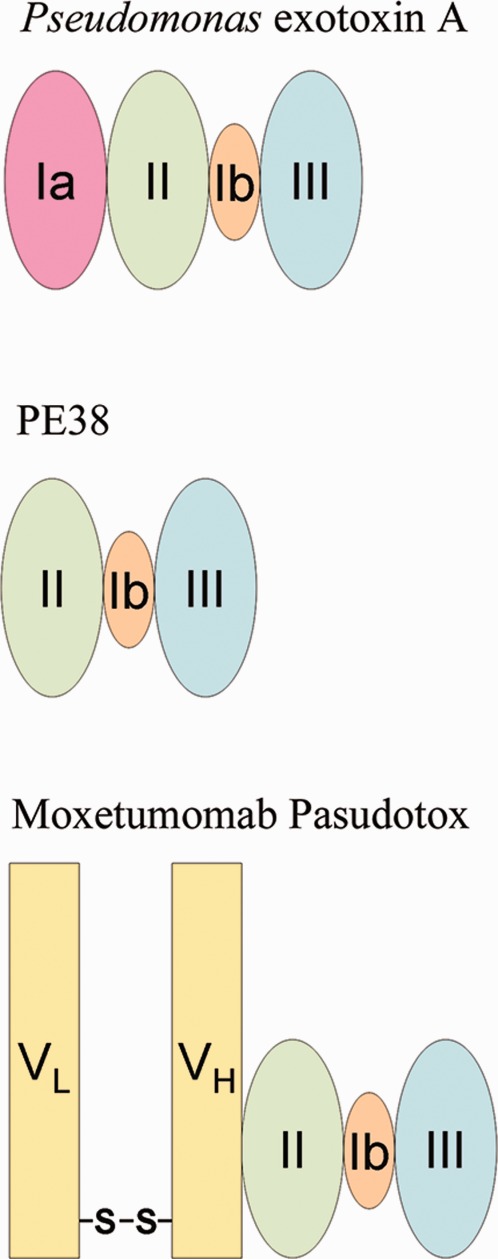
Structure of PE, PE38, and m. pasudotox.The m. pasudotox molecule is composed of the V_H_ and V_L_ portions of an anti-CD22 antibody connected by a disulfide bond and fused to PE38 by a peptide bond to V_H_.

Moxetumomab pasudotox (m. pasudotox, CAT-8015) is a recombinant immunotoxin composed of the V_H_ and V_L_ portions of an anti-CD22 antibody connected by a disulfide bond and fused to a truncated form of Pseudomonas exotoxin (PE38) by a peptide bond to V_H_. M. pasudotox is currently in clinical trials for the treatment of B-cell malignancies.^5^–^12^ The immunoglobulin variable domain is composed of affinity matured V_H_ and V_L_ chains of an anti-CD22 monoclonal antibody, while PE38 contains the PE toxin domains II and III. Domain II has translocation activity while domain III catalyzes the ADP-ribosylation of elongation factor 2, leading to the inhibition of protein synthesis and cellular death.^13^,^14^ The calculated molecular weight is 63,398 Da and the pI is approximately 5. Figure 1 depicts the structure of PE, PE38, and m. pasudotox.

Immunotoxins are typically manufactured using recombinant microbial expression systems. In the case of m. pasudotox, this is accomplished using *Escherichia coli* (*E. coli*) where the two chains are expressed in separate fermentations and recovered as inclusion bodies (IBs). The IBs are combined and refolded to form the active immunotoxin. The refold reaction mixture is then purified to remove process and product related impurities.

Industrial protein refolding presents a number of unique challenges. In particular, low refold titers and high levels of misfolded protein and aggregates commonly encountered in refold reaction mixtures often prove exceedingly difficult to purify, leading to low yield and high cost of goods. To overcome these challenges, refolding conditions undergo extensive development, including the use of high throughput screening techniques to identify the optimal buffer components.^13^,^14^ In addition, advanced control strategies including the use of dissolved oxygen to monitor product quality^15^ and fed batch dilution^16^,^17^ are also employed.

In this work, we discuss the development and scale-up of a commercial immunotoxin refolding and purification process. Challenges with the existing clinical process are explained. The development strategy is outlined and data is presented describing improvements made to inclusion body recovery, solubilization, clarification, protein refolding, and capture chromatography. Results from the 950 L commercial scale refolding process are provided, demonstrating a greater than fivefold improvement in volumetric productivity compared to the clinical process.

## Materials and Methods

### Chemicals and recombinant protein

Chemicals for analytical methods and lab scale experiments were obtained from VWR Scientific (West Chester, PA) and Sigma (St. Louis, MO). The m. pasudotox V_H_-PE38 chain or V_L_ chain were expressed in separate fermentations as IBs using recombinant *E. coli* strains.

### Inclusion body recovery

Cell mass was harvested with a disc-stack centrifuge. Harvested cells were re-suspended with Tris buffered saline (50 mM Tris, 100 mM NaCl, pH 7.4) before homogenization. Cells were lysed by continuous high pressure homogenization. IBs were recovered by continuous centrifugation with a disk stack centrifuge. IBs were washed by re-suspension and mixing with a dilute Triton X-100 solution in Tris EDTA (TE; 50 mM Tris, 20 mM EDTA, pH 7.4). Washed IBs were recovered by centrifugation. Additional washes were performed by re-suspension and mixing of IBs with TE buffer only. The final washed IB slurry was aliquoted and stored frozen.

### Solubilization and clarification

V_H_-PE38 and V_L_ IB solubilization starting concentration were 0.28–0.30 g V_H_-PE38 and 0.06–0.07 g V_L_ per kg refold. IBs were combined in a 1:1 molar ratio of V_H_-PE38 to V_L_ and diluted with TE buffer. Diluted IBs were mixed with 5–6 volumes of IB solubilization buffer (50 mM ethanolamine, 8 M urea, 0.5 M arginine, 2 mM EDTA, 10 mM DTT or DTE) and mixed for 90 min at room temperature with constant stirring. Solubilized IBs were clarified by filtration through increasingly tighter depth filters connected in series (filter grade C0HC followed by filter grade X0HC, Millipore, Billerica, MA). The clarified filtrate was concentrated by tangential flow filtration to 1/10th of the final refold volume using a 5 kDa molecular weight cutoff (MWCO) ultrafiltration membrane.

### Protein refolding

M. pasudotox was refolded by a 10-fold dilution of the clarified and concentrated inclusion body filtrate into pre-chilled (2–8°C) refolding buffer (50 mM ethanolamine, 1 M arginine, 2 mM EDTA, 1.0 mM oxidized glutathione) with mixing. The refold reaction was allowed to proceed for 48–72 h at 2–8°C. Following refolding the reaction mixture was warmed to room temperature in preparation for concentration and buffer exchange. The refold solution was first concentrated by tangential flow filtration with a 10 kDa MWCO membrane and then diafiltered with 10 volumes of Fractogel TMAE equilibration buffer (20 mM phosphate, pH 7.4). Bench scale refolding was performed in jacketed glass vessels with a nominal volume of 5 L. Pilot scale refolding was performed in a jacketed stainless steel tank with a nominal volume of 250 L. The manufacturing scale clinical and commercial refolding processes were performed in jacketed stainless steel tanks with a nominal volume of 950 L.

### Fractogel TMAE capture chromatography

The concentrated and diafiltered refold solution was loaded onto a Fractogel TMAE column (EMD Biosciences, Billerica, MA) equilibrated with TMAE equilibration buffer. After loading, the column was first washed with TMAE equilibration buffer, followed by a dilute Triton solution (20 mM phosphate, 0.1% Triton X-100, pH 7.4) and buffered sodium chloride (20 mM phosphate, 100 mM NaCl, pH 7.4). The product was eluted from the column with 20 mM phosphate, 200 mM sodium chloride, pH 7.4. After elution the column was either regenerated with 2 M NaCl and then sanitized with 1 N sodium hydroxide (clinical process) or stripped with 3 CV 50 mM ethanolamine, 0.5 M arginine, 2 mM EDTA, 8 M urea, 10 mM DTT, pH 9.3, followed by regeneration with 2 M NaCl and sanitization with 1 N sodium hydroxide (commercial process).

The Fractogel TMAE column cycling study was carried out as follows. Three purification cycles were performed on the column followed by a blank elution cycle. This sequence was repeated two more times for a total of nine purification cycles. Step yield, product quality, HETP and asymmetry data were collected as a function of cycle number.

### Intermediate and polishing chromatography

After the initial Fractogel TMAE capture step, m. pasudotox was further purified by hydroxyapatite (HA), HIC and Q Sepharose HP (QHP) chromatography. Purifications were performed as follows. HA chromatography was operated in flow-through mode. The Fractogel TMAE capture step product was loaded without further adjustments onto a HA column (Bio-Rad Laboratories, Hercules, CA) equilibrated with pre-equilibration buffer (400 mM phosphate, 200 mM sodium chloride, pH 7.4) and equilibration buffer (20 mM phosphate, 200 mM sodium chloride, pH 7.4). The product was collected in the flow-through fraction. The HA product was subsequently diluted at a 1:1 ratio with a high salt load preparation buffer (20 mM phosphate, 1.2 M sodium sulfate, pH 7.4) and loaded onto a HIC column equilibrated with equilibration buffer (20 mM phosphate, 0.6 M sodium sulfate, pH 7.4). After loading, the column was washed with equilibration buffer. Product was eluted from the column with a 20 CV linear gradient from 0 to 100% elution buffer (20 mM sodium phosphate, pH 7.4). After elution the HIC product pool was buffer exchanged into 10 mM Tris, pH 8.0, using tangential flow filtration with a 10 kDa MWCO membrane.

The final polishing step has been described in detail in Linke et al. (2012).^18^ Briefly, the diafiltered HIC product was loaded onto a QHP column (GE Healthcare, Piscataway, NJ), pre-equilibrated with elution buffer (10 mM Tris, 1 M sodium chloride, pH 8.0, buffer B) and equilibrated with equilibration buffer (10 mM Tris, pH 8.0, buffer A). After loading, the column was first washed with buffer A and then washed with 35% buffer B. The product was then eluted from the column with a 10 CV linear gradient from 35 to 55% buffer B. For both the HIC and QHP columns, fractionation and offline HPLC analysis was performed as part of the clinical process, whereas *A*_280_ collection criteria were utilized in the commercial process.

### Analytical methods

M. pasudotox analytical methods for purity including SDS-PAGE, high performance anion exchange (IEC), size exclusion chromatography (HPSEC) and bioactivity have been described in detail in Linke et al. (2012).^18^ Fragment levels were measured by reversed phase, high performance liquid chromatography using a 2.0 mm × 50 mm analytical Agilent PLRP-S column with an Agilent 1200 HPLC system.

Refold titers in clinical manufacturing lots were measured by liquid chromatography/mass spectroscopy on an LTQ Velos/ETD mass spectrometer in conjunction with a Waters ACQUITY UPLC system. Reversed-phase chromatography separations were performed on a Michrom 8 µm, 4,000 Å, 1.0 mm × 50 mm column using mobile phase A of 0.1% TFA in water and mobile phase B of 0.1% TFA in acetonitrile. 7.0 µL of refolding samples were injected in duplicate between the calibration curves and reference standards. Mass spectroscopy data were collected at an *m*/*z* range of 300–2,000. Refold titers in commercial manufacturing lots were measured by high performance anion-exchange chromatography using a 2.0 mm × 250 mm analytical Dionex SAX-10 column with an Agilent 1200 HPLC system. The column was equilibrated at a flow rate of 0.2 mL/min with 20 mM sodium phosphate buffer, pH 7.4. Refold samples were diluted fourfold with HPLC-grade water and 100 µL injected onto the column. Refolded m. pasudotox was eluted with a linear gradient from 0 to 310 mM NaCl in 20 mM phosphate, pH 7.4. The eluted protein was monitored by ultraviolet (UV) absorbance at 280 nm.

Host cell protein (HCP) levels were measured using an ELISA assay with antibodies raised against HCP obtained from the *E. coli* host strain used to express m. pasudotox. *E. coli* DNA levels were determined by qPCR using *E. coli* DNA specific primers. Endotoxin levels were measured with the Charles River Endosafe Kinetic Chromogenic LAL Test System. IR spectra were measured on a SpotLight 400 FTIR microscope (Perkin Elmer, Waltham, MA).

### Cryo Transmission Electron Microscopy (CryoTEM)

CryoTEM was performed by NanoImaging Services (La Jolla, CA). Three microliters of inclusion body sample were applied to a cleaned grid and immediately vitrified in liquid ethane. Vitreous ice grids were transferred into the electron microscope using a cryostage that maintains the grids at a temperature below −170°C. Electron microscopy was performed using an FEI Tecnai T12 electron microscope, operating at 120 keV.

## Results and Discussion

### Process overview and challenges

**Figure 2:**
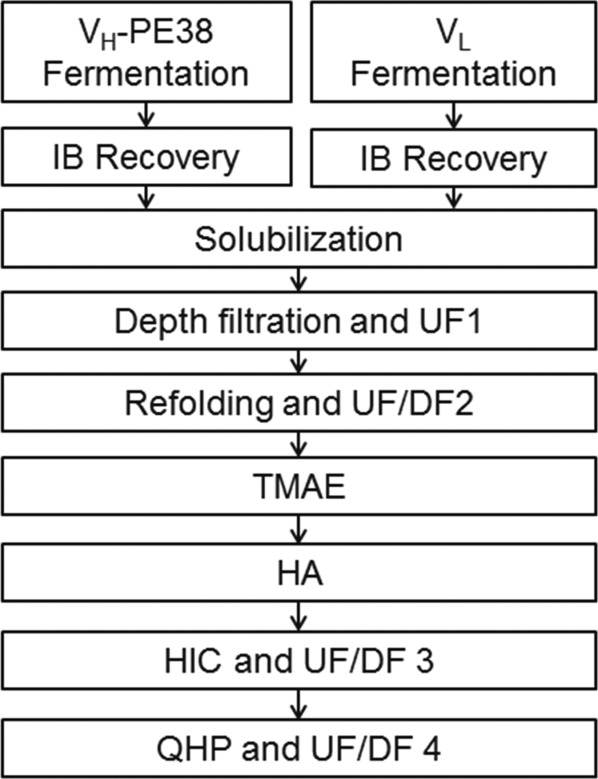
Process flow diagram for the manufacture of m. pasudotox (clinical and commercial).

Like many biotherapeutics, m. pasudotox has seen several iterations of manufacturing processes introduced to meet the higher material and quality requirements that are normally encountered during drug development. In this work, we will be discussing two iterations, a clinical process and the commercial process designed to replace it. Figure 2 shows a flow diagram for both the clinical and commercial processes. Even though the overall process scheme remains unchanged, several changes were introduced to the commercial process to improve performance and reduce cost of goods while maintaining product quality. Table1 provides a summary of major changes to the recovery and purification processes as well as what challenges were addressed by each. The subsequent sections are dedicated to a detailed discussion of development and implementation of these changes.

**Table 1:** Summary of Major Changes to Inclusion Body Recovery, Refolding, and Purification

Unit Operation	Clinical Process	Commercial Process	Reason for Change
Inclusion body recovery	550 bar lysis pressure, six washes	1,000 bar lysis pressure, three washes	Improve facility fit and inclusion body quality
Inclusion body solubilization	pH 10.5	pH 9.3	Decrease deamidation, improve process consistency
Refolding	Batch dilution	Fed batch dilution (4 h addition time)	Improve yield
Capture chromatography	Sodium chloride column regeneration	Urea, arginine, and DTT column regeneration	Improve column lifetime and yield
HIC and QHP chromatography	Fractionation and offline HPLC fraction analysis	*A*_280_ pooling criteria	Simplify the process and improve consistency

### Inclusion body recovery

High quality IBs are critical to the success of a refolding process. Georgiou and Valax define IB quality based on two parameters^19^: The first is purity, or in this context the amount of contaminating material incorporated within the IB aggregate; the second is separability, or the degree to which IBs can be separated from materials having a similar sedimentation coefficient when subjected to a centrifugal field. While the choice of strain and fermentation conditions usually has the greatest impact on IB quality, the recovery process also plays a crucial role in meeting these requirements. Typically, the IBs are released from the cells using a high pressure homogenizer. They are then subject to a series of washes to remove contaminating materials such as adsorbed cellular debris.

The commercial IB recovery process centrifugation steps were developed using the principle of equivalent settling area.^20^,^21^ Accordingly, parameters for scale-up of a disc stack centrifuge are related through the following equations

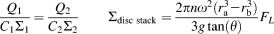
where *Q*_1_ and *Q*_2_ are the volumetric feed rates, *C*_1_ and *C*_2_ are correction factors accounting for non-ideal flow and Σ_1_ and Σ_2_ are the equivalent settling areas at two different scales, *n* is the number of discs in the stack, *ω* is the angular velocity, *r*_a_ is the inner radius of a disc, *r*_b_ is the outer radius of a disc, *F*_L_ is a correction factor dependent on disc spacing, *g* is the acceleration due to gravity and *θ* is the disc angle. The commercial process homogenization step was developed to ensure efficient cell disruption as determined using optical microscopy and particle sizing (results not shown). Table2 summarizes operating and performance parameters for the clinical and commercial processes. Scale-up of the commercial process from the 100 L pilot scale fermentation to 4,500 L scale resulted in similar process performance as shown in Table2.

**Table 2:** V_H_-PE38 Inclusion Body Recovery Operating and Performance Parameters

	Process		
Parameter	Clinical	Commercial	Commercial
Fermentation scale (L)	600	100	4,500
Centrifuge type	Disc stack	Disc stack	Disc stack
Lysis pressure (Bar)	550	1,000	1,000
No of IB washes	6	3	3
*Q*/Σ (m/s)	2.9 × 10^−7^	3.3 × 10^−9^	3.3 × 10^−9^
Overall solids yield* (%)	12.3	4.9	5.9
Overall product yield*,† (%)	–	37.5	42.1

* Measured from end of fermentation to final IB fraction.

† Measured by RP-HPLC.

**Figure 3:**
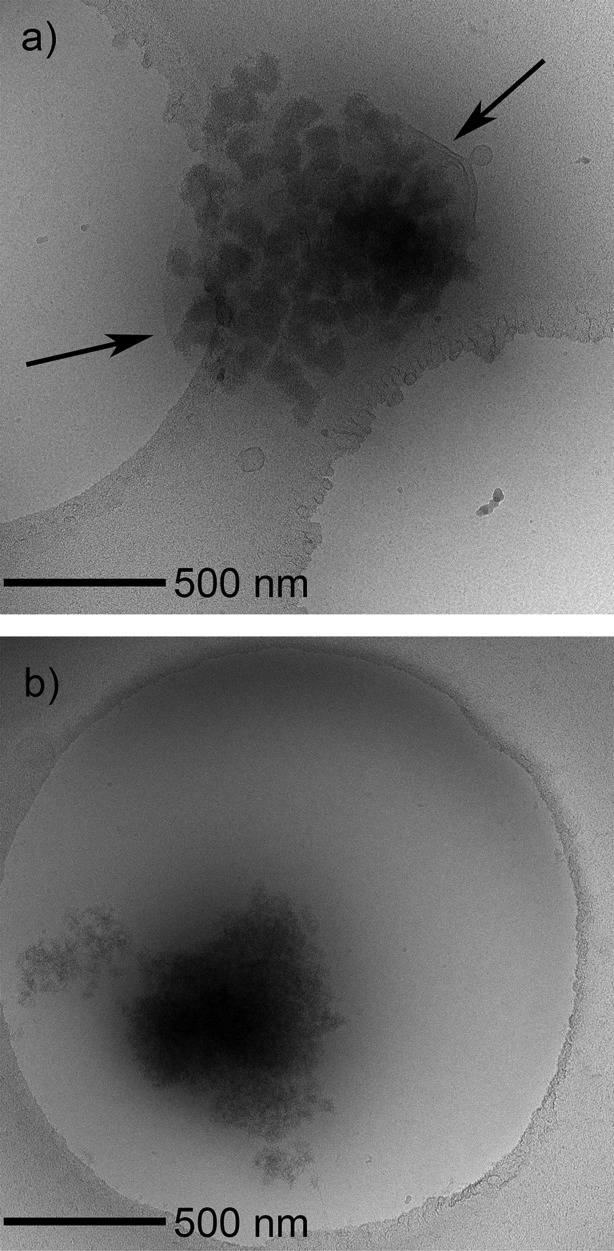
Cryo TEM analysis of V_H_-PE38 IBs obtained from (a) 600 L fermentation scale clinical process and (b) 100 L fermentation scale commercial process.Arrows indicate possible cellular debris associated with the clinical IBs. Circular openings in the TEM grid can be seen in both images.

IB quality was assessed by TEM, FT-IR and SDS-PAGE analysis. Figure 3a shows a representative V_H_-PE38 inclusion body obtained from the clinical fermentation and recovery process. As can be seen from the figure, the IBs are small protein aggregates that appear to be loosely held together, possibly by associated cellular debris (indicated by the arrows). The presence of cellular debris in the clinical IBs was confirmed by FT-IR. After solubilizing protein using a chaotrope, the remaining insoluble material was determined to have some similarity to a previously published IR spectrum for bacterial peptidoglycan.^22^ Cellular debris most likely contributed to the higher percent solids yield observed in the clinical IB recovery process (Table2), and poor filterability as described in the subsequent section. Based on the TEM and FT-IR results, we postulate that the low homogenization pressure used in the clinical recovery process led to incomplete cell disruption. As a result, IBs remained trapped in and could not be separated from large fragments of cellular debris and membranes. The use of six IB washes may have exacerbated this problem by enriching for the largest particles, which under the conditions of incomplete disruption represented IBs trapped within cellular debris fragments and not free IBs. The small size of any free IBs combined with six IBs washes would tend to deplete free IBs from the solids fraction through loss in the centrate fraction.

**Figure 4:**
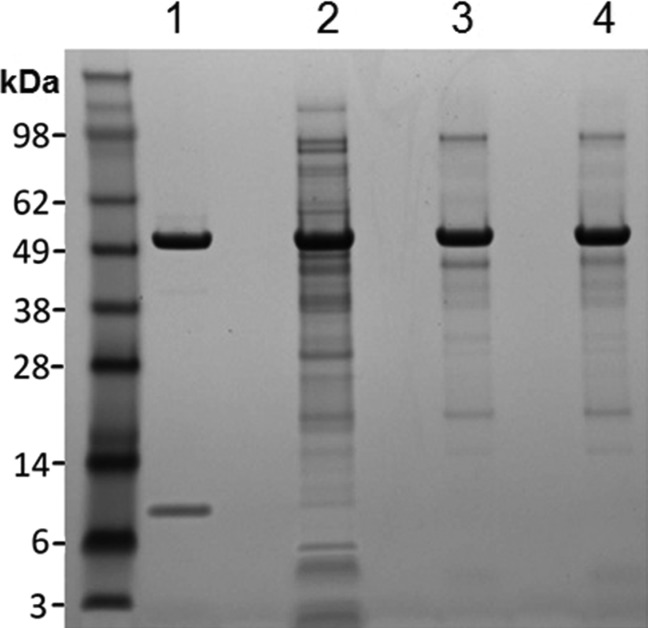
SDS-PAGE analysis of V_H_-PE38 IBs obtained from clinical and commercial fermentation and recovery processes.Lane 1, m. pasudotox standard; Lane 2, clinical 600 L fermentation and recovery process; Lane 3, commercial 100 L fermentation and recovery process; Lane 4, commercial 4,500 L fermentation and recovery process.

Conversely, TEM analysis of an IB from the commercial process piloted at 100 L scale is shown in Figure 3b. Compared to the clinical process and reflecting improvements in fermentation, the aggregate is much larger and more uniform, facilitating sedimentation in a centrifuge. Reflecting improvements in IB recovery as a result of efficient cell disruption and removal of contaminating materials by washing and centrifugation, the IB is largely free of cellular debris and membranes. SDS-PAGE analysis was consistent with results obtained by TEM and FT-IR. Figure 4 shows a gel image of clinical, 100 L scale commercial, and 4,500 L scale commercial IBs. The commercial IBs showed markedly higher purity as reflected in the large number of additional bands seen for the clinical IBs.

### Inclusion body solubilization and refolding

Also consistent with high quality IBs, as shown in Table3, the commercial IBs demonstrated much lower turbidity when solubilized in the presence of a chaotrope. This result correlates with the TEM and FT-IR analysis of the clinical IBs in which cellular debris and membranes were observed. As a result of the lower turbidity, the solubilized commercial IBs demonstrated superior filterability when clarified in a depth filter train. As is shown in Table3, while both the clinical and commercial IBs could be clarified to <5 NTU, an order of magnitude greater filter capacity could be achieved with the commercial IBs. This results in a much lower filter area requirement and reduced processing time.

**Table 3:** Comparison of Solubilized IB Clarification Performance

Inclusion Body Process Starting Material	Turbidity of Solubilized IB Solution (NTU)	Turbidity of Post-C0HC Depth Filtration (NTU)	Turbidity of Post-X0HC Depth Filtration (NTU)	X0HC Depth Filter Capacity (L/m^2^)
Clinical	773	613	3.4	48
Commercial	18.7	12.7	4.0	>450

Perhaps the most important improvement in the commercial process was the control strategy for product deamidation. For the clinical process, most likely due to the presence of partially lysed cells, cellular debris and membranes as discussed above, solubilization at pH 10.5 was required to ensure efficient IB dissolution. However, this condition led to the formation of an inactive deamidated species that had to be controlled as part of the purification process using ion exchange chromatography with fractionation and offline HPLC analysis.^18^ This approach led to low overall yield and high yield variability as will be discussed in subsequent sections.

**Figure 5:**
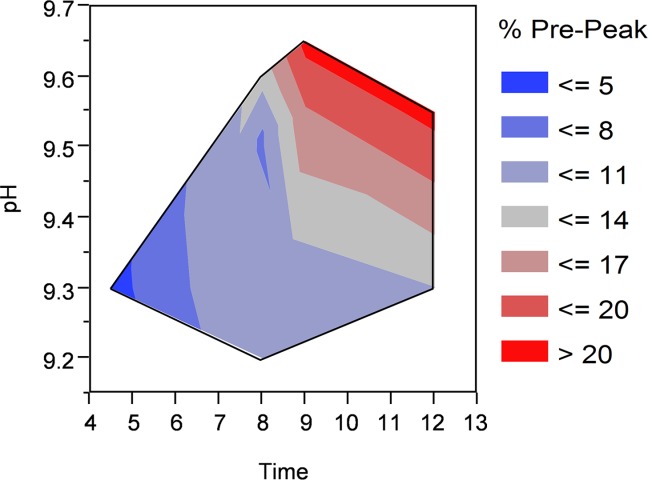
Solubilized m. pasudotox deamidation as a function of pH and time.Deamidation is measured by IEC pre-peak level after refolding and capture chromatography.

With the introduction of the commercial IBs, a control strategy was implemented that prevented formation of large amounts of deamidated m. pasudotox by lowering the reaction rate and limiting the extent of reaction. Figure 5 shows product deamidation as measured using an ion exchange HPLC assay as a function of solubilization pH and time. The level of deamidation increases dramatically at higher pH and longer time, exceeding 20% pre-peak under the conditions tested in this set of experiments. As a result, the commercial process utilized pH 9.3 and a maximum of 12 h to solubilize IBs and limit the extent of the deamidation reaction. Interestingly, despite the slightly higher pH of 9.4 used for the refolding buffer compared to solubilization, there is very little deamidation after the solubilized mixture is added to the refolding buffer (data not shown). The site of deamidation has previously been determined to be Asn-358.^18^ Based on these results, it is likely that this residue is largely buried and protected from solvent exposure upon folding.

To better understand deamidation behavior, a mechanistic model was developed to describe deamidation reaction kinetics. Base catalyzed deamidation of Asn residues has been shown to proceed via a cyclic imide reaction mechanism to produce aspartyl (Asp) and iso-Asp residues.^23^–^27^ To model this system, we followed an approach similar to that of Pace et al.^28^ where the reaction is first order with respect to protein concentration:

First order rate equation

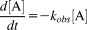


Integrated form

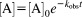
where [A] is the concentration of amidated m. pasudotox, [A]_0_ is the initial concentration, *t* is time and *k*_obs_ is the observed first order rate constant. To capture the potential for specific base catalysis due to hydroxide ions and general base catalysis due to buffer species, Kirsch and Notari^29^ employed a variable rate constant allowing for parallel reaction pathways. We took a similar approach where

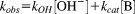
where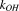 is the specific base catalysis rate constant, [OH^−^] is the hydroxide ion concentration,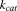 is the general base catalysis rate constant, and [B] is the general base concentration.

**Figure 6:**
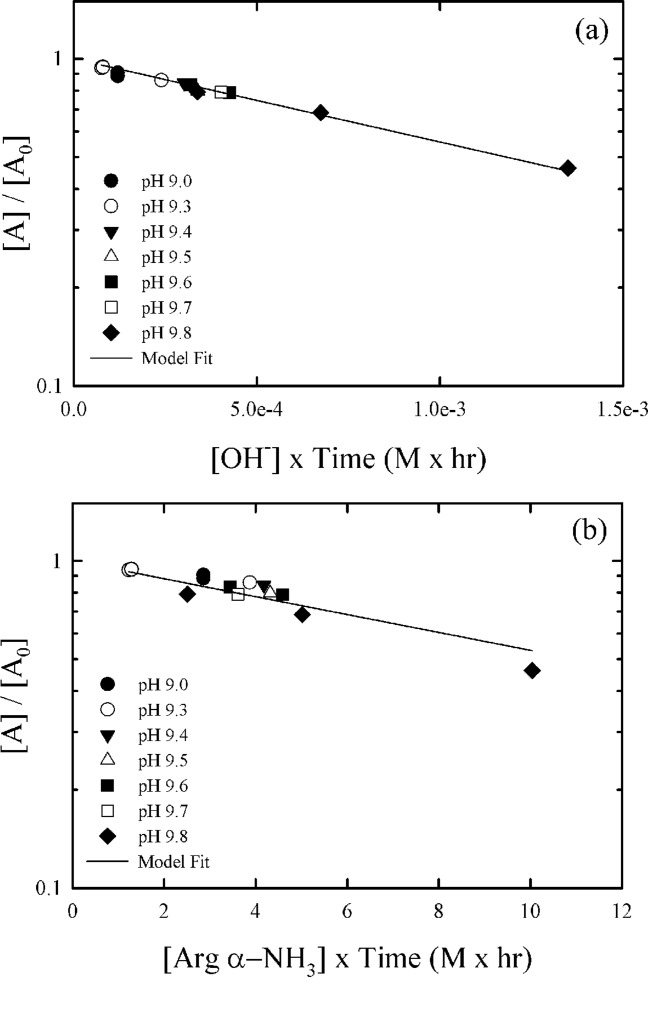
Modified first order kinetic plots of solubilized m. pasudotox deamidation for the two limiting cases where specific base catalysis (a) and general base catalysis (b) dominate.Deamidation is measured by IEC pre-peak level after refolding and capture chromatography. Data shown in both panels was obtained from the same set of experiments.

In a first order kinetic plot, ln([A]/[A]_0_) versus time results in a linear relationship where the slope is used to determine the rate constant. We can take a similar approach by plotting ln([A]/[A]_0_) vs. time×[OH^−^] or time×[B] where the slope can be used to find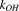 or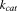 provided one reaction pathway dominates. Figure 6 shows modified first order kinetic plots for these two limiting cases. For this system, the general base is assumed to be the unprotonated form of the α-NH_3_ group on arginine, which has a p*K*a of 9.04. The total arginine concentration is 0.5 M and the unprotonated concentration is 0.25 M at the p*K*a. Potential catalysis due to ethanolamine and Tris were neglected as they are present in much lower concentrations than arginine. Catalysis due to the α carboxylic acid group and guanidinium group of arginine were also neglected as the α carboxylic acid group is far from its p*K*a and the guanidinium group is fully protonated in the pH range of our experiments. The specific [OH^−^] and general [α-NH_3_] base concentrations were assumed constant over the course of an experiment as the solutions were well buffered.

Within the conditions examined in this study, we were not able to distinguish between specific and general base catalysis. In both limiting cases a good fit was obtained for the same data set, with *R*^2^ = 0.98 for specific base catalysis and *R*^2^ = 0.87 for general base catalysis. The values of *k*_OH_ and *k*_cat_ obtained from regression analysis were 5.8 × 10^2^ M^−1^ h^−1^ and 6.3 × 10^−2^M^−1^ h^−1^, respectively. The interpretation is confounded by the fact that both hydroxide ion and general base concentrations are a function of pH over the pH range used in our experiments. To better elucidate the reaction mechanism, additional studies would be required varying the buffer concentration in addition to the pH. While the better fit obtained for specific base catalysis would tend to suggest this reaction pathway plays a more important role, the uncertainties described above prevent this conclusion. Moreover, literature states that asparagine deamidation may occur principally due to general base catalysis under conditions used in this study.^26^,^30^ Nonetheless, from a practical standpoint the specific base catalysis model would provide an excellent predictive capability of m. pasudotox deamidation as a function of pH for purposes of process optimization and control, so long as other parameters and buffer concentrations are maintained constant.

The rate of deamidation measured in our experiments appears to be consistent with those reported by Li et al.,^31^ who investigated asparagine deamidation for a set of model peptides. First order rate constants for solution reactions observed in their detailed studies ranged between 1 × 10^−5^ s^−1^ and 5 × 10^−5^ s^−1^ at pH 9.0, with the exception of AcGQNGG which was much more reactive. The models developed in our study predict a *k*_obs_ value between 2 × 10^−6^ s^−1^ and 4 × 10^−6^ s^−1^ at pH 9.0. This is approximately an order of magnitude lower than *k*_obs_ values reported by Li et al. The difference is to be expected, though, since Li et al. performed their experiments at 70°C and our work was conducted at room temperature.

Another important improvement to the commercial refolding process was implementation of fed batch dilution refolding. In this technique, the addition of solubilized protein at a gradual controlled rate results in higher yields as lower concentrations of folded and unfolded protein are maintained over the course of the addition as compared to rapid batch-dilution refolding.^16^,^17^

Protein refolding yields are usually determined by the competition between folding and aggregation reactions. Refolding of monomeric proteins typically follows first order kinetics for on-pathway formation of the desired product, while undesired off-pathway aggregation reactions can be described by second order or higher kinetics.^32^,^33^ Therefore, suppression of the aggregation pathway relative to proper folding can often be achieved by reducing the protein concentration in solution. However, the success of this approach was uncertain for m. pasudotox, as it can be anticipated that like aggregation, the separate chains coming together to form a dimer will be described by a second order reaction. Therefore, if the reaction orders for the on-pathway and off-pathway reactions are similar, it will be difficult to gain a relative advantage and suppress the off-pathway reaction using a fed batch dilution strategy.

Table4 shows a comparison of batch and fed batch refolding for m. pasudotox. With the exception of addition time, all parameters for the two experiments at the 5 L refold scale were identical. In this case, fed batch addition resulted in a 34% increase in refold volumetric productivity. These results suggest on-pathway dimerization of the V_H_-PE38 and V_L_ chains may not be rate controlling. Rather, steps in the reaction mechanism that are expected to be first order, for example folding of the toxin domain, are likely to be rate controlling under these circumstances. As a consequence, any reduction in the rate of product formation at lower protein concentration is more than compensated for by a greater reduction in the rate of aggregation, leading to a net increase in the formation of properly folded product.

**Table 4:** Comparison of Batch and Fed Batch Refolding Volumetric Productivity at the 5 L Scale

Refolding Volumetric Productivity	Batch Dilution Refolding*,†	Fed Batch Dilution Refolding†,‡
Folded product purified§ per unit volume of refold reaction (mg product/L refold reaction)	42.2, 44.4	59.2, 57.3

* Dilution time: <1 min.

† Results shown for two replicate runs.

‡ Dilution time: 4 h.

§ Measured after TMAE capture chromatography.

**Figure 7:**
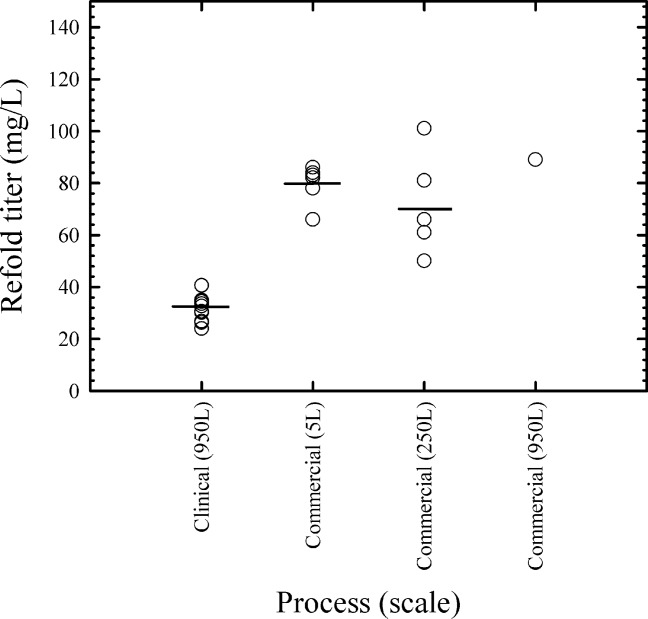
M. pasudotox refolding titer for different processes and scales.

Improvements to the solubilization and refolding process proved to be scalable. Figure 7 compares refolding titers across multiple scales ranging from 5 to 950 L. While there is some scatter in the data as a result of the crude nature of the material, performance across scales for the commercial process was similar. In addition, the increase in refold titer from the clinical to the commercial process was on average 44 mg/L, representing a greater than twofold increase (*n* = 12 for the clinical process and *n* = 13 for the commercial process). It should be noted that the refold titer measurement includes both amidated and deamidated species of m. pasudotox. Moreover, the level of deamidation in the clinical process was 45% (*n* = 12) when measured by IEC pre-peak. As a result, since the deamidation level is <10% for the commercial refolding process, the increase in volumetric productivity of active amidated m. pasudotox is approximately fourfold. This improvement represents the combined effects of higher IB quality, solubilization pH and fed batch dilution.

### TMAE capture chromatography

Following refolding, the reaction mixture is concentrated and diafiltered to facilitate capture on an anion exchange column. The crude nature of the refold reaction mixture, containing a high percentage of aggregated and misfolded protein, led to a severe column fouling problem and corresponding short column lifetime.

**Figure 8:**
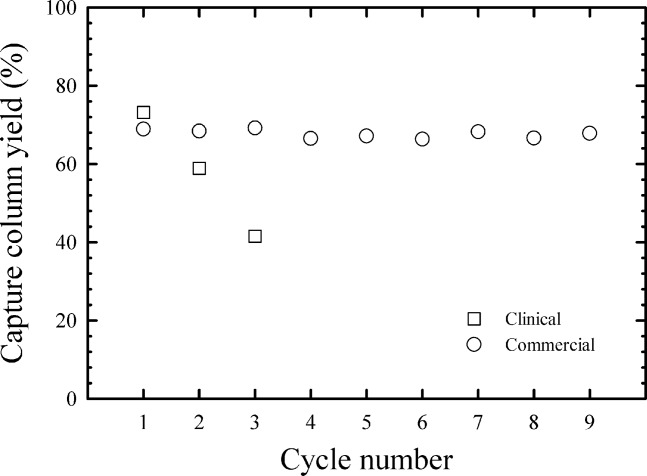
Capture column yield as a function of cycle number.

In the clinical process, the column was regenerated with sodium chloride and then sanitized with sodium hydroxide. This procedure resulted in a large amount of UV absorbing material eluting with the sodium hydroxide sanitization. As it suggests a significant amount of protein remains bound to the column after regeneration with sodium chloride, this situation is undesirable. The strongly bound protein led to a column lifetime problem as shown in Figure 8, where yield for the clinical process drops by 31% after three cycles.

**Figure 9:**
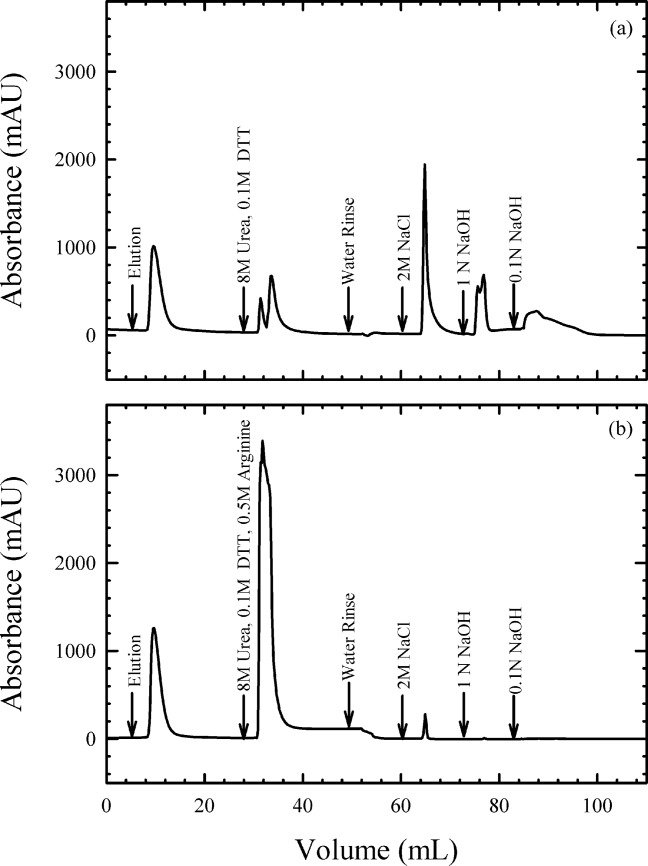
Impact of arginine on capture column stripping performance (a) column stripping with 8 M Urea, 0.1 M DTT (b) column stripping with 8 M Urea, 0.1 M DTT, 0.5 M arginine.

To ensure more complete removal of bound proteins, a stripping procedure was introduced utilizing urea as a chaotrope and DTT as a reductant. Figure 9a shows the *A*_280_ profile obtained for product elution and column cleaning using this stripping procedure. While the introduction of urea and DTT showed a substantial improvement compared to the clinical process, as can be seen from Figure 9a, there is still a significant amount of *A*_280_ absorbing material eluting with the sodium hydroxide sanitization.

This problem was solved using a combination of urea, arginine and DTT. Figure 9b shows the *A*_280_ profile of the final improved stripping procedure. Compared to Figure 9a, sanitization with sodium hydroxide led to very low levels of UV absorbing material eluting from the column. The improved stripping procedure translated into superior column lifetime as demonstrated in Figure 8, where product yield proved to be stable over nine cycles in a resin lifetime study.

The cause of this apparent synergistic effect between urea and arginine is not completely understood. However, we can speculate that similar to protein refolding, arginine may serve to stabilize structures that are partially unfolded which would otherwise tend to strongly aggregate. In this case, rather than preventing insoluble aggregates from forming, the stabilizing effects of arginine appear critical to dissociate and solubilize protein that has already formed insoluble aggregates. Alternatively, the arginine may provide ionic strength that modulates the interaction between charged functional groups on the protein and the ion exchange resin, which, in combination with urea and DTT provides superior removal of bound proteins than urea or salt alone.

### Process performance and product quality summary

Table5 summarizes process performance and product quality of the commercial refolding process at the 250 L and 950 L scale. Analytical results demonstrate that the m. pasudotox product has low levels of process related impurities including HCP, DNA, and endotoxin. Moreover, the control strategy implemented in the commercial process resulted in low levels of deamidated product as measured by IEC HPLC. The scalability of the process is also shown in Table5. The volumetric productivity of the 250 L and 950 L commercial processes were 27.0 and 32.2 mg purified m. pasudotox (drug substance) per liter refold, respectively.

**Table 5:** Process Performance and Product Quality Summary of Commercial Process at 250 L and 950 L Refold Scale

Step	Column Volume (L)	Step Yield (%)	HPSEC Monomer (%)	IEC-Deamidation (% Pre-Peak)	RP-Fragments (%)	HCP (ng/mg)	Endotoxin (EU/mg)	DNA (pg/mg)	Bioactivity (% of Reference)
Commercial (250L)									
Buffer exchanged refold product	–	–	–	–	–	1,443	14,711	–	–
TMAE*	3.2	96.7†	96.9	7.3	–	186.2	44.7	<1	–
HA	1.7	97.2	97.4	6.8	7.9	41.1	2.0	1	–
HIC	1.5	83.4	99.6	5.2	1.3	9.3	0.3	<1	–
QHP	1.6	87.1	99.9	3.2	1.1	3.6	<0.1	<1	104
Commercial (950L)									
Buffer exchanged refold product	–	–	–	–	–	1,593	7,629	30	–
TMAE	31.8	83.6†	97.5	8.8	–	28.0	<48	<3	–
HA*	2.9	98.1	97.1	8.6	8.1	16.3	5.4	<3	–
HIC	6.1	76.8	99.6	7.2	1.9	<3.3	7.8	<4	–
QHP	6.1	72.1	99.5	4.8	1.6	<2.2	<0.004	<3	114‡

* Column cycled twice.

† Yield was determined by IEC titer and *A*_280_ assay. All other yields were determined by *A*_280_ measurements.

‡ Measured after buffer exchange into formulation buffer.

As is also shown in Table5, step yields at the 250 L and 950 L refolding scales were generally similar. Some differences in yield can be attributed to analytical variability. For example, the crude nature of the refolded product prior to capture chromatography is challenging to analyze and results in increased assay variability. However, the QHP column yield difference across scales is due largely to process variability as a result of on-column aggregation (data not shown). While this phenomenon resulted in a decrease in yield, product quality was maintained as the aggregated material was strongly retained on the QHP column and separated from the monomeric product. In the course of laboratory scale studies, it was found the yield loss is correlated with increased temperature. To improve QHP column yield in future manufacturing campaigns, operating temperature will be controlled at a lower set point for this chromatography step.

Finally, Table6 shows a comparison of product yield, yield variability and volumetric productivity for the clinical and commercial processes at 950 L scale. Illustrating the dramatic nature of the improvement a >5× increase in both product yield and volumetric productivity of purified m. pasudotox was demonstrated for the commercial process.

**Table 6:** Comparison of Final Purified Product Yield, Yield Variability and Volumetric Productivity for the Clinical and Commercial Processes at the 950 L Refold Scale

Parameter	Clinical	Commercial
Average final purified product yield* (g)	4.8	30.8
Yield range (g)	2.4–9.5	29.4, 32.1
Yield coefficient of variance	0.49	0.06
Volumetric productivity* (mg product purified/L refold reaction)	4.8	32.2
Number of batches	12	2

* Measured after UF/DF 4.

## Conclusions

**Figure 10:**
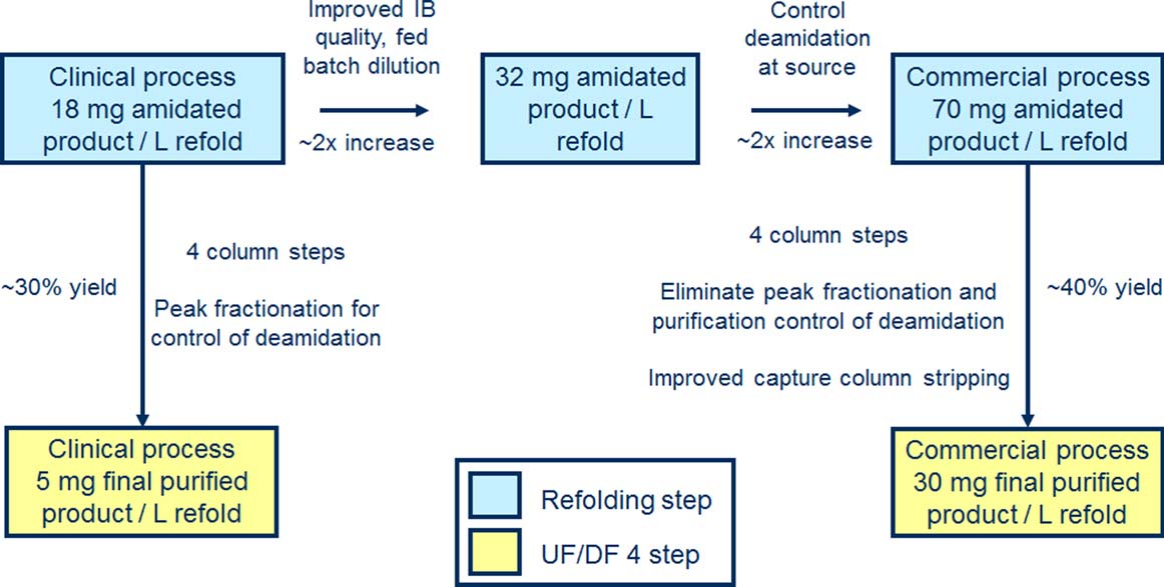
Systematic improvements leading to a greater than fivefold increase in volumetric productivity for the commercial m. pasudotox manufacturing process.

This work presents a case study for development and scale-up of a commercial recombinant immunotoxin refolding process. A systematic approach to process development and understanding resulted in a number of improvements, the cumulative effect of which led to a dramatic improvement in product yield and volumetric productivity compared to the prior clinical process. Changes to the fermentation and inclusion body recovery processes provided higher IB quality and better performance downstream. Formation of an undesired deamidated species was controlled during inclusion body solubilization. Refold titer was increased by 34% through implementation of fed batch dilution. Lastly, a novel combination of urea, arginine and DTT provided efficient capture column cleaning enabling at least nine cycles with no deterioration in performance. The cumulative impact of these improvements on volumetric productivity is shown in Figure 10.

The scalable nature of the commercial process is also illustrated by this work. Results obtained at the 250 L and 950 L refold scales showed similar process performance and product quality. In particular, levels of deamidated m. pasudotox were low and maintained within 5% across scales. Compared to the clinical process it replaces, the commercial process demonstrated a greater than fivefold improvement in volumetric productivity at the 950 L refolding scale.
